# Providing Physical Health Checks for People with Severe Mental Illness in Primary Care in England: An Evaluation of a Locally Enhanced Service

**DOI:** 10.3390/nursrep14040282

**Published:** 2024-12-06

**Authors:** Sheila Hardy

**Affiliations:** Charlie Waller Trust, Newbury RG14 5SJ, UK; sheila.hardy@charliewaller.org

**Keywords:** mental illness, physical health, primary care, annual health check, locally enhanced service, training, practice nurses

## Abstract

**Background/Objectives**: People with a severe mental illness die much earlier than the rest of the population from a preventable physical illness. Annual health checks are a way of assessing the person to then offer the appropriate interventions. Integrated Care Northampton, England used the long-term plan baseline funding allocated to them from the government department that commissions primary care services, to implement a local enhanced service. Their aim was to provide a person-centred physical health check that people with severe mental illness feel comfortable, confident, and able to engage with. **Methods**: Wellbeing Organisation Research Training Hub Northampton were commissioned by Integrated Care Northampton to provide training, support, and evaluate the locally enhanced service. Training was provided by training trainers who then offered one-to-one support to those delivering health checks in practice. Providers of the health checks could also access individual support from Wellbeing Organisation Research Training Hub staff. Patient data were collected via a template that is part of usual practice. Questionnaires were used to evaluate the education of staff, the delivery of health checks, and the impact on people with severe mental illness. **Results**: Training was well received but most of the trainers did not continue in their role. The project was successful in highlighting the physical health needs of people with severe mental illness and monitoring in primary care increased. Though methods were put in place to evaluate the delivery of health checks and their impact on people with severe mental illness, these were not utilised by the service. **Conclusions**: This paper emphasises how difficult it is to implement a new service and evaluate it successfully. Future projects should prioritise measuring the quality of the service.

## 1. Introduction

It is recommended that people with severe mental illness (SMI) receive a physical health check at least once a year [1,2]. This is because life expectancy in this group is reduced by up to 25 years [3,4]. The major cause of death is cardiovascular disease caused by a combination of smoking, unhealthy diets, lack of exercise, and weight gain [5]. Some of the antipsychotic medications used to treat people with SMI have been implicated in raising this risk [6]. Many other physical conditions are also prevalent in this group [7]. Additionally, people with SMI who have an increased risk of self-neglect have the highest excess mortality [8].

In their technical guidance for physical health checks for people with SMI [9], NHS England write that people with SMI are three times more likely to attend emergency care with an urgent physical health need, and those who do attend are then five times more likely to receive an emergency admission. They are also 10 times more likely to be hospitalised because of a physical health condition.

Health services have a legal obligation to make reasonable adjustments to ensure that people with SMI are not disadvantaged compared with the general population in accessing healthcare [10]. In an attempt to tackle this disparity, targets were set in the Mental Health Five Year Forward View [11], and subsequent long-term plan [12] to ensure that at least 60% of people with SMI receive an annual physical health check. This annual standard was raised to 80% for 2023–24.

Data submitted by Northamptonshire to NHS England before the start of this project show that all six of the required elements of the physical health check were administered to 14% of the SMI cohort (less than a quarter of the expected target). When compared to other integrated care systems (ICSs) in the neighbouring area, Northamptonshire ranked in the lowest quartile, completing less than 50% of that achieved by half of their neighbouring sustainability and transformation partnerships (STPs). When the Northamptonshire data regarding the physical health check were analysed by each of its six component parts, the levels of achievement are significantly increased (between 42% and 72%). This suggests that patients with SMI were being monitored (either planned or opportunistically), but not all elements were being considered.

Integrated Care Northamptonshire (ICN), the NHS organisation that plans and delivers health services in Northamptonshire, used the long-term plan baseline funding allocated to them from NHS England to implement a local enhanced service (LES). They set out to provide each primary care centre with enhanced payments for patients who annually receive the required elements of the SMI physical health check. The number of required elements was increased from six to twelve:

1. BMI/waist circumference;
2. Blood pressure and pulse check
3. Blood lipid including cholesterol;
4. Blood glucose test;
5. Assessment of alcohol use;
6. Assessment of smoking status;
7. Assessment of nutritional status, diet, and level of physical activity;
8. An assessment of use of illicit substance/non prescribed drugs;
9. Access to relevant national screenings;
10. Medicines reconciliation and review;
11. General physical health enquiry into sexual health and oral health;
12. Indicated follow-up interventions.

ICN stated that the aim of this LES was to ensure a person-centred service that people with SMI felt comfortable, confident, and able to engage with. The aim of the evaluation was to measure how well this service was delivered.

## 2. Materials and Methods

ICN commissioned Wellbeing Organisation Research Training Hub (WORTH) Northampton to support them in the delivery of the LES. WORTH Northampton is a limited company that provides training and carries out research in the field of wellbeing. WORTH Northampton also commissioned the author to carry out an evaluation of the project over a two-year period. In their latest national guidance for the physical health of people with SMI, NHS England recommend an evaluation of services, which includes patient and staff experience [13]. Data were collected for the author inhouse; this has many advantages, including being cheap and quick, and encourages maximum involvement and participation [14]. Data were obtained through evaluation forms, questionnaires, and extraction from a template in patients computer records.

### 2.1. Participants

The participants of this project included:

1. Trainers delivering the training to practitioners;
2. Practitioners delivering the health checks in primary care and their managers and administrators;
3. People with SMI receiving health checks in primary care.

In addition to the overall aim, the following objectives were set:

○ Staff delivering training and assisting primary care staff will feel prepared and supported in this role;
○ Staff providing the health checks will be educated to understand the needs of people with SMI and know how to deliver the best care for this group;
○ Managers of staff carrying out the health checks will enable them to deliver the best care;
○ Staff providing the health checks will feel supported to carry out the role;
○ There will be a significant increase in the county of each element of the health check completed;
○ There will be a significant increase in the county of all elements of the health check completed;
○ People with SMI attending a health check will be offered help to change any identified unhealthy behaviours;
○ People with SMI will engage in activities to improve their health.

It was anticipated that the intervention could make an impact on the following:

○ Visits to emergency care by people with SMI will be reduced;
○ There will be a reduction in hospital admissions in this group;
○ There will be an increase in measures (blood pressure, BMI, cholesterol, and glucose) that are in a healthy range;
○ More people with SMI will have stopped or reduced smoking, increased their exercise, improved their diet, and reduced their alcohol intake since their health check.

### 2.2. The Intervention

The intervention was developed by WORTH to achieve the objectives. It included education and support.

#### 2.2.1. The Education

Training was via a three-hour face-to-face module. It is based on research that includes a service user perspective of physical health and accessing care. It is divided into bite size pieces so delivery can be split into separate sessions. It was written by a nurse expert in the subject and focusses on preparing primary care practices to offer health checks for people with SMI. The need for follow-up and support after the initial health check is emphasised. It was hoped that participants would feel inspired and encouraged to take on the responsibility, and that any negative attitudes would be dispelled. The specific objectives of the training were for the participant to:

○ Understand how a person is affected by severe mental illness;
○ Know how to work with a person with severe mental illness;
○ Recognise relapse in mental health;
○ Be aware of the physical health issues in severe mental illness;
○ Consider how to make appointments accessible;
○ Be able to monitor physical health in severe mental illness;
○ Appreciate the importance of supporting behaviour change.

Participants were provided with a handout and directed to two websites. Firstly, to a website created by the author of the training, which contains the relevant information and documents to support them in a practical sense to deliver the health checks (https://webeden.co.uk/ (accessed on 26 July 2024)), and to the local WORTH website (https://www.worthwellbeing.co.uk/smi-physical-health-checks/ (accessed on 26 July 2024)). The WORTH website provides written information and videos and links to two external films:

- https://www.southeastclinicalnetworks.nhs.uk/phsmi/—this film covers the main points of the training (albeit more briefly) and gives healthcare professionals the opportunity to revisit areas of interest (accessed on 26 July 2024).
- https://www.youtube.com/watch?v=gw4qt5IbVls—this film provides a service user view of accessing a physical health check (accessed on 26 July 2024).

#### 2.2.2. Delivery of Training

The training was provided as a train-the-trainer (TTT) program. TTT refers to a programme of education where practitioners receive training on a defined subject and instruction on how to train, monitor, and supervise other professionals. A training toolkit was supplied, including a Power Point presentation, participant handout, trainer handout, training schedule, and evaluation forms; this was delivered by the author of the training on behalf of Charlie Waller Trust. Charlie Waller Trust is a charity that provides education to staff in schools, universities, and the workplace. It was anticipated that two nurses would be trained from each of the four primary care networks (PCNs). PCNs are groups of GP practices and other local health and social care services that provide integrated and personalised care for people.

#### 2.2.3. Rolling Out the Training

Training was delivered to practice staff in two ways:

- Small groups in a venue away from the GP practice. Staff could book onto these either online through the WORTH website or by ringing the project administrator.
- Sessions delivered in individual GP practices. These were offered by the trainers to practice managers.

#### 2.2.4. Support for Trainers

Trainers were able to access the CWT trainer for individual advice as part of the TTT delivery. They were able to obtain support from the WORTH project team in the form of action learning sets (via TEAMS). The purpose of the action learning sets was to enable the trainers to learn from each other’s experience and find ways to deal with issues in training and health check delivery. They were also able to contact the WORTH project team with individual queries.

#### 2.2.5. Support for Practitioners Delivering Physical Health Checks

To enhance their willingness to undertake the role and increase the likelihood of providing the same level of care from individual practices, the practitioners delivering the health checks could access advice and support from the trainers and members of the WORTH project by contacting them by email. They could also access information from the WORTH website.

#### 2.2.6. Support for Administrators and Managers Organising Physical Health Checks

Practices received monthly motivational communications with the offer of a ‘Helpdesk’ (WORTH email address included for questions and queries). Training for administrators was offered via TEAMS sessions delivered by a GP and administrator. The training included using a template (Ardens) that codes the clinicians’ interventions, thus enabling accurate measuring. Practice staff could also request one-to-one support.

#### 2.2.7. Addressing System Inequalities

To encourage more people with SMI to attend their health check appointment, we:

- Provided information about health checks for service users and made this available to GP practices, a local mental health charity (MIND) and the county’s mental health service. The information used was adapted from that provided by the national charity Rethink Mental Illness.
- As part of the training, participants were advised of ideas and opinions given by people with SMI about the way health checks and follow up can be delivered.

#### 2.2.8. Advertising the Intervention

We wanted to make sure that practice managers, GPs, nurses, administrators, and other appropriate healthcare workers were aware of the intervention. We wanted them to feel motivated to look after the physical health of people with SMI and feel supported in doing so.

Training courses were advertised on the WORTH website and were bookable through a platform that connects people with events (Eventbrite). GP practice staff were advised how to book training via the website in email communications from ICN. Additionally, a separate flyer with all the relevant information was attached to the emails. Email communications continued throughout the year to inform practices of their achievement and provide an opportunity to ask questions or book onto more training.

### 2.3. Measures

Measures were put in place to evaluate the education of staff, the delivery of health checks, and the impact on people with SMI.

#### 2.3.1. Trainers

Trainers were asked to complete an evaluation form following their training, and a questionnaire six months later.

#### 2.3.2. Staff Delivering Health Checks

- Staff providing the health checks were asked to complete an evaluation form following their training, and a questionnaire six months later. The trainers were asked to keep a record of any issues brought up by the staff and how they were resolved.
- The managers of staff carrying out the health were asked to complete a questionnaire.

#### 2.3.3. Impact on Numbers of People with SMI Receiving Health Checks and Follow Up

To find out the percentage of people with SMI who had elements of the health check completed and been offered help to change any identified unhealthy behaviours, we planned to measure data captured on the Ardens template.

#### 2.3.4. Impact of the Service on the Health of People with SMI

The purpose of the physical health checks is to improve the health and reduce the mortality of people with SMI. It is not possible in the short time scale of a service evaluation to measure whether the service had an impact on mortality, but some measures and indications of health could be looked at by carrying out searches using the appropriate codes.

If possible, to find out whether hospital admissions and visits to emergency care by people with SMI have reduced during the time period, we asked if data could be requested before and after the project from the two hospitals in the county.

#### 2.3.5. Impact on People with SMI’s Experience of the Service

To find out the experience of people with SMI who attended a health check and subsequent follow up, we designed a short patient questionnaire. This included questions about how they were invited for their health check, their experience of the service offered, and whether they felt it motivated and/or supported them to make positive changes to their health behaviour. We wanted reception staff at GP practices to assist patients to complete this questionnaire on paper after their appointment. We were aware of how busy staff are and how this may be a burden on them, so we suggested that two patients from each of the 69 GP practices would be enough to give us a picture of the patient experience.

### 2.4. Analysis

Descriptive analysis using Excel was used to compare patient outcomes before and after the intervention. Thematic analysis was employed for staff questionnaires.

## 3. Results

### 3.1. Training the Trainers

An advertisement to take part in the project as a trainer was emailed to all primary care practices in Northamptonshire. In total, nine nurses took part in the training. Four nurses attended a face-to-face training session in May 2022. Of these, one decided she did not wish to participate, and two were unable to continue because they could not be released from their clinical duties. Further advertisements were emailed, and the WORTH team approached nurses who they were aware had an interest in mental health. Three nurses attended a face-to-face training session in August 2022. Two more nurses were unable to attend this training so took part in an online session in October 2022. One nurse from the first session and two from the second went onto to deliver the training to healthcare professionals. Due to health reasons one trainer had to cease taking part. By the end of the project, there were two trainers still actively involved in training delivery.

### 3.2. The Trainers

In order for us to understand how they were feeling about their role, the trainers were asked to answer questions via an online survey following their training. Seven of the nurses completed it.

The main reasons cited for taking part in the project were a passion for mental health, a desire to improve outcomes for people with mental health problems, and to increase their skills.

The nurses were asked what help or support they needed to feel confident in delivering the training. Six of the respondents stated they felt confident with level of support that was in place. In response to the question asking how they feel about supporting health care professionals in primary care to deliver health checks for people with severe mental illness, one was very confident, and the rest were reasonably confident because there was someone they could ask for help if they need it.

One trainer completed the survey six months after their initial training. They told us about the help or support they received that enabled them to deliver the training. They said:

*The action learning sets were helpful as they could discuss issues with their peers and the trainer.*

*There was enough administration support to enable them to deliver the training.*

They described some of their experience supporting health care professionals in primary care to deliver health checks for people with severe mental illness, stating:

*They felt confident as they were well prepared.*

*Most of the healthcare professionals appeared motivated to learn about delivering health checks.*

### 3.3. Staff Delivering Health Checks

#### 3.3.1. Pre-Training

We wanted to find out how health checks are currently delivered in primary care and to offer help in organising them if staff felt this would be helpful. An email was sent to all practice managers. It offered assistance from WORTH and asked the managers to complete a short questionnaire. Two replies were received. Both practices stated that they invited patients with SMI for a designated health check appointment, one provided by a specified member of staff and the other by any member of the clinical team. One practice had completed clinical and administrator training provided by WORTH and were using the resources provided. The other practice was aware of the training and resources and was making plans to organise attendance and to use them.

#### 3.3.2. Training Attendance

Thirty training sessions were delivered in various venues in the county. One hundred and two healthcare practitioners attended. It was calculated by ICN that 70% of practices in the county were represented.

#### 3.3.3. Feedback from Training

Trainees attending the sessions were given evaluation forms and asked to score themselves against the statements (which were the training objectives) between one and five, with one being disagree and five being agree. These forms had been reviewed by experts in the field in past projects. Thirty-four people completed the forms, which were added to an Excel spreadsheet by an administrator. Their average scores are detailed in Table 1 below. It can be seen from the scores that following the training, attendees had a good understanding of how a person is affected by SMI and their physical health issues and felt confident to carry out health checks. They were less confident in working with a person with SMI and recognising when they experience a relapse in their illness.

Trainees were asked for comments regarding their perceived knowledge and confidence following the course.

*‘The course was interesting, but I haven’t worked in mental health so am quite unsure.’*

*‘Enabled me to feel more confident with SMI reviews.’*

Trainees were also asked to score the quality of the training in the same way. It can be seen from Table 2 that the attendees felt the course was relevant and well presented. The aims were explained clearly, it was pitched at the right level, and attendees learnt a lot.

Trainees were asked for comments regarding the quality of the course.

*‘Lovely Trainer, really explained so well, thank you.’*

*‘Very relevant, excellent. Thank you.’*

*‘Really interesting and informative session. Really love the trainer’s passion and knowledge.’*

*‘I feel that there should be training for reception staff to help engage people and to book review appointments.’*

*‘Good set of training, interactive and kept interest. Lots of opportunities to discuss.’*

*The trainer was very helpful and informative.*

### 3.4. Support for Staff Delivering Health Checks

None of the healthcare practitioners taking part in the training contacted their trainer for support or advice, nor did they complete the survey sent to them six months after training. Therefore, we do not know what issues they encountered or how they were resolved. Nor can we report whether they felt supported in practice to provide the health checks and the appropriate follow up.

There were three queries to the WORTH inbox, and all were offered a one-to-one consultation to answer their query.

The WORTH website can still be accessed for supporting information. The addition of training videos has been planned but this was not available at the time of writing this article.

### 3.5. Support for Administrators

All practices signed up to Ardens Manager with permission for data extraction. Forty-five administrators attended one of the two training sessions (one for each computer system used by GP practices) provided. Thirty practices were given one-to-one support. Observations noted during these contacts were:

- Some practices were unaware of the 12-point checks and the incentive scheme;
- Few administrators were aware of how to check their year-to-date achievement;
- Physical health checks carried out by the county’s mental health service were often incomplete;
- Non-achieving codes were mistakenly added to computer systems.

The ‘Helpdesk’ offer of support was popular and many follow up questions were received.

### 3.6. Case Studies

We wanted to highlight good practice by writing case studies regarding organisation of health checks and outcomes for patients. We approached individual practices and sent out an email request offering a telephone interview. Only one GP practice responded.
**Case Study: Whole Practice Approach****Aim and rationale:** The aim of this GP practice approach was to give people with severe mental illness the best care they can have and get to know their population with SMI. They wanted to make relationships with people with SMI and not just tick boxes. They felt this was possible as the SMI population in their practice is small (90 out of 10,000 patients).**Preparation and organisation:** After finding out about the SMI project from ICN in October 2022, the practice staff agreed that this was worthy of their time. They set up a team to organise the delivery of physical health checks for people with SMI consisting of the practice manager (PM), a care coordinator (CC), and a GP. They had support from the rest of the partners and team to take this on. The CC attended the online WORTH course for administrators. The GP attended the WORTH face-to-face training for healthcare professionals and has watched all the available films and videos. Patients with SMI are invited to see a healthcare assistant or nurse prior to their health check for routine measurements and blood test, meaning that the GP has this information at the health check appointment. The PM sets up regular clinics for the GP to carry out the health checks. In the first year the appointments were 45 min long but were reduced to 30 min in the second year; less time was needed as the relationships with patients had been built. People living in care homes are visited there. The CC books the health check appointments for people with SMI. Once a month the team meet for a morning or afternoon to discuss some of the patients with SMI and check the SMI register. Individual tasks regarding patients are dealt with daily as needed.**The health check** The CC is present at the health check. He records the action plan. The GP performs a full physical examination and acts upon any findings as appropriate. Any measures or blood tests missed are performed. She also provides suitable lifestyle advice and makes referrals as necessary (e.g., weight management and stop smoking groups, opticians, and dentist).**Following the health check** The CC sends a letter to the patient advising them of what was discussed in the health check. He then rings the patient to discuss the action plan and support them with any behaviour change identified. The GP sees patients again when she has introduced or changed a medication, and when a clinical issue is identified during the check.**Overcoming challenges:** Some people with SMI have not responded to the invitation for a health check. The CC rings them to encourage them to come. On one occasion a patient has walked out of a consultation but did return for another appointment, which went well. The focus has been on building trust with this group. The team feel that the remuneration provided by ICN does not cover the cost of providing the service they are giving.**Outcomes:** Outcomes have been positive:Patients have got to know the GP and CC. They appear more open and relaxed when attending.Several safeguarding issues have been identified and dealt with.Concerning symptoms have resulted in 2 week wait referrals being made.Other medical conditions have been identified, for example, a person with aortic stenosis whose family were also screened.Cardiovascular risks factors have been identified and treated as appropriate.The team delivering this care feel proud and satisfied in what they are doing.**Recommendations to others:**Providing this service needs to be a team effort. A care coordinator is necessary, and the rest of the practice team need to be supportive.Adequate time needs to be given to carry out the health check in order to perform all the required activities.

### 3.7. Impact on Numbers of People with SMI Receiving Health Checks and Follow Up

**Figure 1:**
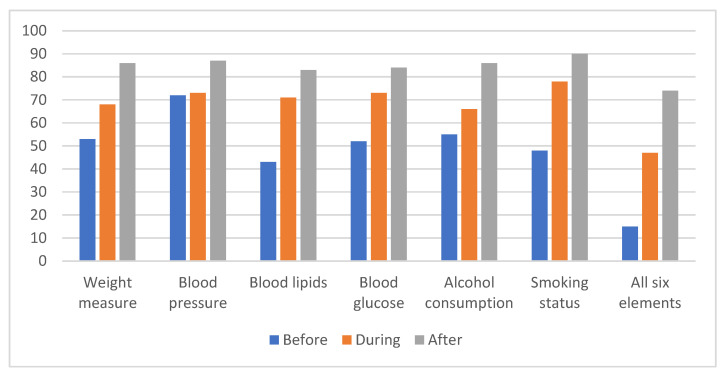
SMI Physical health checks: Comparison of percentage measurement of six core elements, before, during and after project.

According to the Arden’s data, only 55% of patients with SMI were recorded as having had an annual health check, yet achievement of individual elements of the check was higher. We compared the percentage measurement of the six core elements of the physical health checks for people with SMI. It can be seen from Figure 1 that there has been a steady increase.

**Figure 2:**
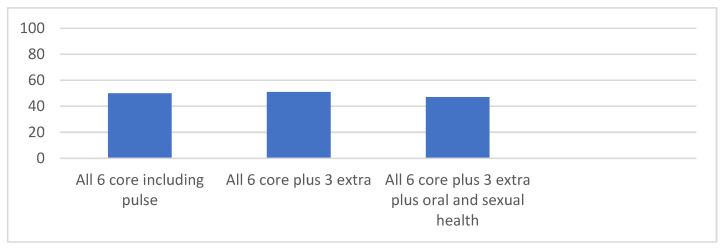
Percentage of patients receiving all six core checks plus extra.

Figure 2 shows the percentage of patients who received more than six elements of the health check. Only 50% of patients with SMI had had their pulse rate measured. Enquiry about oral and sexual health is low.

**Figure 3:**
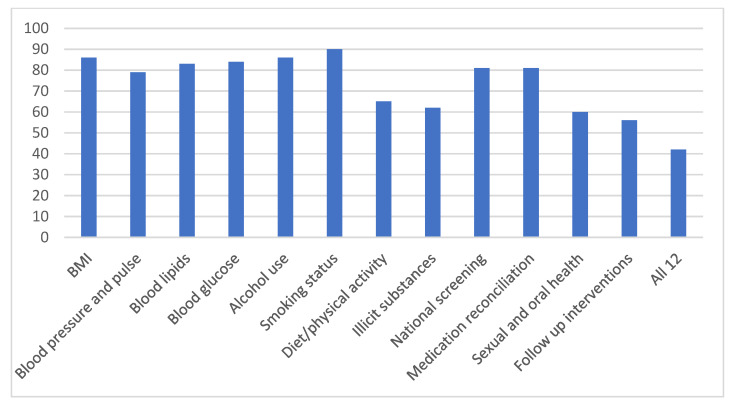
Percentage of the elements of the physical health check recorded in the last 12 months (n = 5431).

Figure 3 shows the percentage achieved of each of the 12 elements that are required by the LES. A high level of achievement was reached in the clinical measures and screening. Recording of oral and sexual health, use of illicit substances, and diet and physical activity status were lower. Fifty-six percent of patients with SMI who had a recognised need were offered the appropriate follow up intervention.

Table 3 shows the percentage of patients in the last 12 months who were offered an intervention following an assessment of need. It is important to remember that patients who have a need but did not receive the measurement will not be included. Interventions for diabetes and smoking scored very highly. Scoring for sexual health and oral health is low, and less than half the patients were offered interventions for substance misuse and blood pressure medication.

### 3.8. Impact of the Service on the Health of People with SMI

It was not possible to analyse measures and indications of the health of people with SMI (as described in the method section) as the data were not provided. Neither could we find out whether hospital admissions and visits to emergency care by people with SMI reduced during the time period for the same reason.

### 3.9. Impact on People with SMI’s Experience of the Service

**Figure 4:**
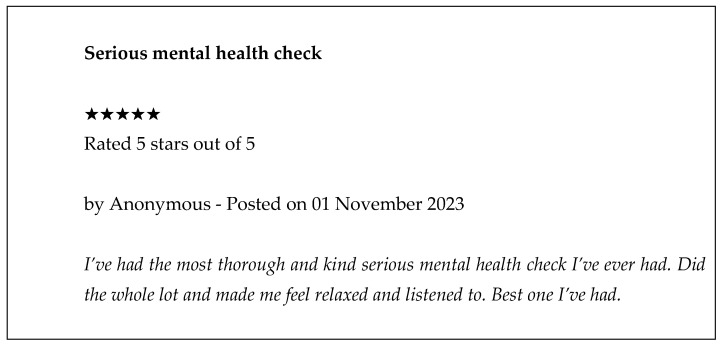
Example of online feedback to NHS site.

We were unable to find out the experience of people with SMI who attended a health check and subsequent follow up, as it was perceived that asking the GP practice staff to assist two of their patients with SMI to complete a paper questionnaire following a health check would be too burdensome. Instead, practice staff were asked to send patients a link to an online questionnaire, despite our advice that this would not yield a good response. We do not know how many practices sent out the link. There was no response at all. The only opinion we received from a patient with SMI was an example of online feedback to one GP practice (Figure 4).

### 3.10. Work Beyond the Project

Further funding from the Population Health Management programme has been secured to continue work with practices via the motivational communications, administrator training, and one-to-one support.

## 4. Discussion

The aim of this project was to ensure a person-centred service that people with SMI felt comfortable, confident, and able to engage with. To assist in this aim, WORTH set out to provide training and support. The trainers that took part in the education felt confident and well-supported by WORTH to carry out their role, but most did not continue. Health Education England report that those involved in education and training are increasingly reporting feeling undervalued and have insufficient time due to increasing service pressures [15]. Nurses taking on the role of trainers need to be given the time and backing from their employers; however, a systematic review found that organisational austerity affects education and expecting nurses to use personal resources is a concerning trend [16]. Future projects like this should consider a way to ensure that nurses are given the appropriate time and support as part of the requirements to be trained as a trainer. Incentivising the trainers’ employers with a financial reward or merit could be considered.

All GP practices were advised of the project details and asked what WORTH could do to assist them to carry out physical health checks for people with SMI. There was little response to this offer and practices did not ask for assistance until later when they needed help to reach the targets. Understanding why practices did not initially engage will help to put the appropriate support in place in future. Elvey et al. explain that creating a system in which the structures that govern primary care can help drive improvements requires staff working in primary care to see themselves as active rather than passive participants in organisational change and have trusting relationships between practices [17].

The clinical training was well received, and attendees felt confident to deliver physical health checks for people with SMI. However, none of the attendees asked for the support or advice that was offered. Nor did they tell us, when asked, how they were managing to deliver health checks in practice and if they were given the appropriate time and assistance by their employers to do this. Therefore, we are unable to report on how well the clinics are being managed in practice. It would be helpful for future projects to understand why healthcare staff did not seek support. A scoping review of common barriers to primary care engagement found that limited time and capacity was a factor that prevented engagement [18].

Only 55% of people with SMI were recorded as having a health check in the past year, yet there was a much higher rate of the individual elements being carried out. This suggests that many patients with SMI have not received a health check but have had the individual elements measured opportunistically. We cannot report whether this is because they were not invited for health check or if they had been invited and did not attend, as this information has not been given.

The level of assessment of each required element of the health check has increased, which is very encouraging, but the offer of some interventions is low. It is not in the scope of this evaluation to look at the quality of the interventions offered but these should be considered. Without the required appropriate intervention, the health check has no benefit to the health and mortality of a person with SMI [9]. Continued monitoring and feedback should encourage an increase in the offer of interventions [19].

It is disappointing that there has been no feedback from patients as without this we have no indication regarding the quality of the health checks being delivered or in fact whether the project has achieved its aim of ensuring a person-centred service that people with SMI feel comfortable, confident, and able to engage with. Future projects need to prioritise collection of patients’ views.

Many of the agreed measures were not carried out because the evaluation team were reliant on the commissioning organisation to provide the data. NHS England assert that internal staff may lack the time and objectivity to take part in a joint approach, which may explain why [14].

Since the start of this project, there has been an increase in the monitoring of the physical health of people with SMI, but more time and resources are needed to fully embed the service and make a positive difference to patients. This judgment is echoed by the Health Foundation, who advocate that implementation needs to be handled carefully and the expectations of NHS England and its national partners about the speed of change need to be measured and realistic [20].

## 5. Conclusions

This LES was set up to provide physical health checks for people with severe mental illness in primary care in England. It has been successful in highlighting the physical health needs of people with SMI and monitoring in primary care has increased. However, the level of some of interventions to improve physical health are low. Future projects should include measuring the quality of the service and providing enough time and resources to enable success.

## Figures and Tables

**Table 1:** Feedback from trainees: impact of training.

Training Objectives	Average Score (n = 34)
I understand how a person is affected by severe mental illness	4.3
I know how to work with a person with severe mental illness	3.6
I can recognise relapse in mental health	3.7
I am aware of the physical health issues in severe mental illness	4.3
I know how to make appointments accessible	4.1
I am able to monitor physical health in severe mental illness	4.1
I appreciate the importance of supporting behaviour change	4.2

**Table 2:** Feedback from trainees: quality of training.

Training Qualities	Average Score (n = 34)
Aims explained clearly	4.8
Taught me a lot	4.6
Relevant	4.6
Pitched at the right level	4.7
Well presented	4.9

**Table 3:** The percentage of patients in the last 12 months who were offered an intervention following an assessment of need.

Intervention	Number of Patients Included	Percentage Receiving Intervention
Weight management	3247	73
Blood pressure: lifestyle	807	69
Blood pressure: drugs	819	49
Glucose: at risk	578	71
Glucose: diabetes	561	87
Alcohol	335	64
Smoking	1800	97
Substance misuse	339	36
Oral health	3349	18
Sexual health	3226	12

## Data Availability

Data are contained within the article.

## Public Involvement Statement

No public involvement in any aspect of this service evaluation.

## Guidelines and Standards Statement

This manuscript was drafted against the Best Practice in the Ethics and Governance of Service Evaluation Guidelines for evaluators and commissioners of evaluation in health and social care, Available online: https://nhsevaluationtoolkit.net/resources/best-practice-guidelines-in-ethics-and-governance-of-service-evaluation/ (accessed on 31 July 2024).
